# Creep Behavior and Its Influencing Factors in High-Entropy Superalloys: A Molecular Dynamics Simulation Study

**DOI:** 10.3390/ma19020233

**Published:** 2026-01-07

**Authors:** Kangning Han, Qiuju Wang, Yaxin Zhu, Shulin Yuan, Changwei Wang, Shuang Liang, Lv Zhao

**Affiliations:** 1 Department of Engineering Mechanics, School of Aerospace Engineering, Huazhong University of Science and Technology, Wuhan 430074, China; 15930773616@163.com (K.H.); yxzhu2006@hust.edu.cn (Y.Z.); yslgsoo1@hust.edu.cn (S.Y.); wangchangwei@hust.edu.cn (C.W.); lvzhao@hust.edu.cn (L.Z.); 2 AVIC Guiyang Wanjiang Aviation Electricalmechanical Co., Ltd., Guiyang 550018, China; 3 Hubei Key Laboratory of Engineering Structural Analysis and Safety Assessment, Wuhan 430074, China

**Keywords:** high-entropy superalloys, creep mechanism, lattice misfit, dual phase, molecular dynamics

## Abstract

In aero-engine applications, turbine blades operate under high-temperature and high-pressure thermomechanical cyclic loading conditions, which demand exceptional mechanical performance. High-entropy superalloys, characterized by a stable dual-phase γ/γ′ microstructure, have emerged as promising candidates for high-temperature structural materials due to their superior creep resistance. In this study, the creep behaviors of high-entropy superalloys are systematically investigated using molecular dynamics simulations, exploring the effects of stress, temperature, γ/γ′ lattice misfit, and γ′ volume fraction on creep deformation mechanisms. The results show that both stress and temperature significantly influence creep behavior, with temperature exerting a more dominant effect. As the applied stress increases, the dominant creep mechanism evolves from atomic diffusion to dislocation nucleation and motion, eventually leading to phase transformation. Additionally, the γ/γ′ lattice misfit and γ′ volume fraction are found to critically affect the alloy’s creep resistance. Specifically, creep resistance initially increases and then decreases with increasing lattice misfit magnitude, while a negative misfit yields better performance than a positive one. Moreover, increasing the γ′ volume fraction enhances the alloy’s ability to resist creep deformation. Microstructural analysis and atomic diffusion data further reveal that the creep resistance of high-entropy superalloys is closely associated with the structural stability of the γ/γ′ dual-phase system. These findings provide useful insights for optimizing the high-temperature performance of high-entropy superalloys through microstructural design.

## 1. Introduction

The demand for gas turbines and aero-engines has increased dramatically in recent decades, leading to increasingly stringent performance requirements for superalloys operating in high-temperature and high-pressure environments [1,2,3]. Conventional Ni-based superalloys, while widely used, are costly and involve complex processing routes [4]. Consequently, the development of cost-effective, high-temperature-resistant structural materials has become a strategic priority in the field of advanced aero-engine manufacturing.

High-entropy superalloys (HESAs) represent a newly developed branch of high-entropy alloys (HEAs). Owing to their γ/γ′ dual-phase microstructure and high-temperature properties comparable to those of Ni-based superalloys—combined with superior cost-effectiveness—HESAs are emerging as promising candidates for high-temperature applications. Among the key properties required for such applications, creep resistance is particularly critical, as it determines a material’s ability to withstand prolonged exposure to elevated temperatures [5]. Creep is a time-dependent deformation process characterized by a gradual increase in strain under stress levels below the material’s yield strength. It occurs in three distinct stages: primary, steady-state, and tertiary creep [6]. Primary creep, also known as transition creep, exhibits an initially high creep rate that decreases owing to the strain hardening phenomenon. This stage usually lasts for a relatively short period. Steady-state creep follows with a constant creep rate. In this process, the creation of new dislocations and annihilation of dislocations reached dynamic equilibrium, reflecting a dynamic balance between strain hardening and recovery. This process lasts for a long time, and it accounts for the majority of a material’s service life. Tertiary creep is significantly characterized by an accelerated increase in creep rate due to the damage nucleation and accumulation, such as the formation and evolution of voids and microcracks, which reduces the effective cross-sectional area and increase in local stress concentrations. Ultimately, this process culminates in the final rupture or failure of the material.

Extensive research has been conducted on the creep behavior and mechanisms of Ni-based superalloys [6,7,8,9] and high-entropy alloys (HEAs) [10,11]. Ni-based superalloys exhibit excellent creep resistance, which is primarily attributed to their γ/γ′ two-phase microstructure. In contrast, single-phase HEAs typically show poor creep resistance at high temperatures [12]. Despite the structural similarities between HESAs and Ni-based superalloys, systematic studies on the creep behavior of HESAs remain limited. As a result, the fundamental creep mechanisms in HESAs are not yet fully understood, which poses a significant barrier to their broader development and application in high-temperature environments. In Ni-based superalloys, the volume fraction of γ′ phase and the lattice misfit of the γ/γ′ microstructure significantly affect their creep resistance [13,14]. By adding γ′-forming elements (e.g., Ti, Al, and so on), the volume fraction of γ′ phase can be increased, thereby narrowing the γ channels, raising the critical Orowan stress required for dislocation motion within them, and enhancing the creep strength of the alloy [15]. For example, Mukherji et al. [16] investigated the creep properties of Ni-based superalloys with different γ′ phase contents at 1100 °C/(140–210) MPa, and they demonstrated that Ni-based superalloys with up to 58% γ′ phase content exhibited longer creep lifetimes than those with 55%. However, it is worth noting that a higher volume fraction does not always lead to better performance. Indeed, it has been demonstrated that Optimal creep resistance is typically achieved when the γ′ phase constitutes 60–70% of the alloy [13]. Excessive γ′ particles would have a negative effect on the creep properties due to particle coarsening and embrittlement. Lattice misfit, defined as the relative difference between the lattice parameters of the γ and γ′ phases, also plays a significant role in creep behavior. The misfit (δ) is calculated as follows [17]:

(1) $$\delta =\frac{2\left(\alpha_{\gamma^{\prime }}-\alpha_{\gamma }\right)}{\alpha_{\gamma^{\prime }}+\alpha_{\gamma }}$$

where $a_{\gamma }$ and $a_{\gamma^{\prime }}$ are the lattice parameters of the γ and γ′ phases, respectively. Commercial Ni-based superalloys typically demonstrate a negative lattice misfit at room temperature, which progressively decreases with rising temperature [18,19]. Harada et al. reported that a higher absolute value of lattice misfit (|δ|) significantly contributes to the enhancement of the alloy’s creep resistance [20]. The interfacial lattice misfit between the γ and γ′ phases has been identified as a critical factor in determining the formation and configuration of dislocation networks. The spacing D of networks can be estimated as follows [15,21]:

(2) $$D=\frac{\left|\vec{b}\right|}{\left|\delta \right|}$$

where $\overset{\to }{\left|b\right|}$ is the magnitude of the Burgers vector. It can be seen that a larger lattice misfit results in a smaller D, leading to denser dislocation networks and stronger interfacial stress fields, which effectively impede dislocation penetration into the γ′ phase, thereby improving the alloy’s creep properties. Within an optimal range, the γ/γ′ interface maintains coherency with low interfacial energy, ensuring interface stability. However, excessive lattice misfit can destabilize the microstructure by causing significant γ′ distortion, reducing creep resistance. Therefore, optimizing both the γ′ phase volume fraction and the γ/γ′ lattice misfit is essential for achieving superior creep performance in superalloys.

Given the microstructural similarity between Ni-based superalloys and HESAs, it is hypothesized that both the γ′ phase volume fraction and lattice misfit may also play crucial roles in determining the creep resistance of HESAs. While extensive experimental and computational investigations have been conducted on the creep behavior of Ni-based superalloys and HEAs [16,22,23,24,25], there remains a significant gap in the systematic understanding of creep mechanisms in HESAs. This study aims to comprehensively investigate the creep mechanisms in HESAs, with particular emphasis on elucidating the effects of lattice misfit and γ′ phase volume fraction on their creep resistance. Among various HESA systems, AlCoCrFeNi-based alloys are particularly promising due to their excellent mechanical properties and thermal stability. As such, they serve as representative candidates for exploring creep resistance in relation to γ′ volume fraction and lattice misfit. The findings in this work will provide valuable insights for designing HESAs with advanced high-temperature performance.

## 2. Simulation Model and Methodology

**Figure 1:**
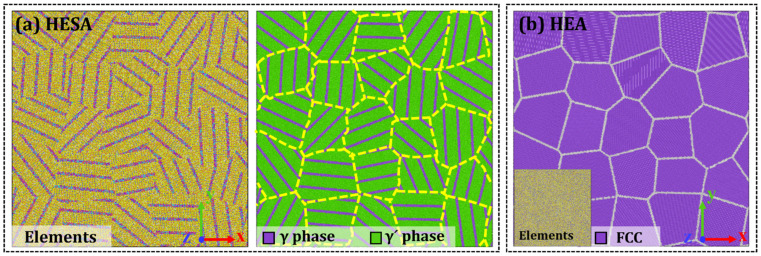
Atomistic model of nanocrystalline: (**a**) HESA and (**b**) HEA.

The γ/γ′ dual-phase microstructure plays a critical role in determining the creep deformation behavior of Ni-based superalloys [13]. The γ′ phase typically exists as three-dimensional cuboidal precipitates embedded within the γ matrix. However, incorporating such cubic precipitates into the simulation would require an excessively large atomic model, rendering long-term creep simulations computationally prohibitive. To address this challenge and improve computational efficiency, the complex three-dimensional γ/γ′ dual-phase structure is simplified into a two-dimensional lamellar configuration in this study. Figure 1 illustrates the polycrystalline atomic model developed for an HESA, with a corresponding HEA model provided for comparison. This model preserves the essential γ/γ′ two-phase structure, ensuring that its creep behavior remains representative of high-entropy superalloys HESAs. Variations in the composition of the two phases directly influence the strength and lattice parameters of each phase, thereby affecting the alloy’s overall mechanical performance [26]. According to some experimental tests [27,28], the matrix phases in both the HEA and HESA were precisely formulated with a composition of Ni_33.4_Al_6.7_Cr_22.2_Fe_9.2_Co_28.5_, while the γ′ phase in the HESA was characterized by a composition of Ni_65.9_Al_12.1_Cr_8_Fe_9_Co_5_ and a volume fraction of 81%. The lattice misfit between these two phases was determined to be 0.1402%. The HEA model was generated by constructing an FCC lattice followed by random substitution of atoms according to the target composition, reflecting the characteristic chemical disorder of HEAs. For the γ/γ′ HESA, the γ matrix was initialized as an FCC Ni lattice and the γ′ phase as an L1_2_ Ni_3_Al lattice, after which Fe, Co, Cr, and Al atoms were substituted into Ni or Al sublattices based on the experimentally determined γ and γ′ compositions. Each polycrystalline model consists of 25 grains with an average grain size of approximately 10 nm. The polycrystalline configurations were generated using a Voronoi-based tessellation procedure implemented via an in-house code developed by the authors, in which randomly distributed grain nuclei were used to partition the simulation cell into grains with realistic shapes and grain boundary networks. After tessellation, each grain was assigned an independent crystallographic orientation. The atomic lattice corresponding to the γ or γ′ phase was then mapped into the respective grain region, followed by the removal of atoms with unphysical proximity near grain boundaries to ensure clean interfaces. The final models, with overall dimensions of 50 × 50 × 1.4 nm, were periodic in all three spatial directions.

**Figure 2:**
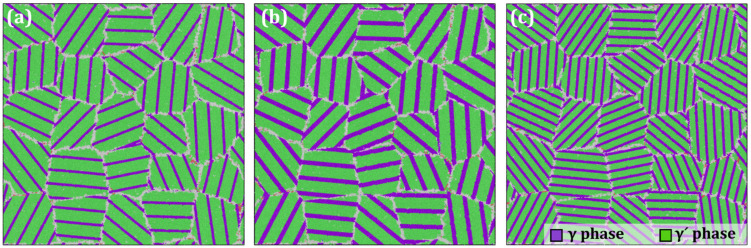
Polycrystalline HESA models with γ′ phase volume fractions of (**a**) 81%; (**b**) 72% and (**c**) 70%.

Building on the HESA model shown in Figure 1, we systematically adjusted the elemental compositions within the precipitate and matrix phases to create a series of polycrystalline HESA models with varying lattice misfits. These models are designed to elucidate the influence of the γ/γ′ two-phase lattice misfit on the creep behavior of HESAs. To prepare each system for creep simulation, energy minimization was first performed using the conjugate gradient (CG) method. Following minimization, the system underwent a short annealing process consisting of two sequential relaxation steps of 50 ps each with a constant time step of 2 fs, allowing grain boundaries to thermally relax before the subsequent NVT and NPT equilibration steps. The first relaxation step employed the NVT ensemble to stabilize temperature, while the second one used the NPT ensemble to equilibrate both temperature and pressure. After these steps, the system reached a stress-free state with minimal energy at a constant temperature, thereby making it suitable for the subsequent loading phase. To describe the atomic interactions, we employed the embedded-atom-method potential developed by Farkas [29] (2020) for the Fe–Ni–Cr–Co–Al high-entropy alloy family. This potential has been validated against key material properties, including equilibrium lattice constants, cohesive energies, elastic constants, mixing enthalpies, defect formation energies, and generalized stacking-fault (SF) energies for Fe-, Ni-, Cr-, Co-, and Al-rich compositions. It can accurately reproduce γ/γ′ phase stability, chemical short-range ordering, and high-temperature diffusivity in AlCoCrFeNi-based HEAs and HESAs. Considering that the Ni-based superalloys typically operate in the temperature range of 600–1200 °C, four representative temperatures (500 K, 750 K, 1000 K, and 1400 K) were selected for the simulations. To accelerate creep deformation within the limited timescale of molecular dynamics simulations, an external uniaxial stress ranging from 0.5 to 4.5 GPa was applied [6]. The selected stress range enables the investigation of stress-dependent creep behavior and mechanism transitions within a consistent MD framework. Loading was applied using the NPT ensemble, with zero pressure maintained in the x and z directions and a constant uniaxial tensile stress imposed along the y direction. The entire creep simulation was conducted over 200 ps, which is enough to model the creep in the molecular perspective [6,30,31]. The time step was set at 1 fs. All simulations were performed using the renowned MD simulator LAMMPS [32,33], and post-processing of the simulation data was carried out using the visualization tool OVITO (3.8.5) [34]. Furthermore, we constructed additional polycrystalline HESA models with different volume fractions of the γ′ phase by precisely controlling the sizes of the precipitate and matrix regions, as illustrated in Figure 2. These models, with γ′ phase volume fractions of 81%, 72%, and 70%, respectively, are specifically tailored to investigate how variations in the γ′ phase volume fraction affect the creep behavior of HESAs. Representative LAMMPS input scripts related to model construction and creep simulations, together with the corresponding interatomic potential files and representative post-processing scripts for creep strain and MSD analysis, have been made publicly available through an open access repository.

To quantitatively evaluate the creep resistance of the alloys, the atomic diffusion coefficient (D) is employed as a key metric to characterize the creep behavior of nanopolycrystalline materials under various temperature and stress conditions. The diffusion coefficient is calculated based on the mean-squared displacement (MSD) of atoms, using the following equation [35]:

(3) $$D=\frac{1}{6t}\langle {\left|r_{t}-r_{0}\right|}^{2}\rangle$$

where *t* is the simulation time, and $r_{t}$ and $r_{0}$ are atomic positions at time t and the initial time, respectively. The coefficient of 1/6 assumes isotropic diffusion, implying that atomic movement occurs equally in all three spatial dimensions. In the present simulation, atomic diffusion takes place not only along grain boundaries (GBs) but also within the grain interior. Consequently, the overall diffusion coefficient *D* is composed of two components: the grain boundary diffusion coefficient *D_GB_* and the lattice diffusion coefficient *D_L_*.

## 3. Results and Discussion

### 3.1. Creep Responses of HESAs

This section focuses on the influence of temperature and applied stress level on the creep behavior of HESAs, as well as the underlying deformation mechanisms. All simulations presented herein are based on a representative HESA model with a γ′ volume fraction of 81% and a γ/γ′ lattice misfit of 0.1402%. These parameter values are selected to ensure a stable dual-phase microstructure while capturing the critical features of creep deformation under service-relevant conditions.

**Figure 3:**
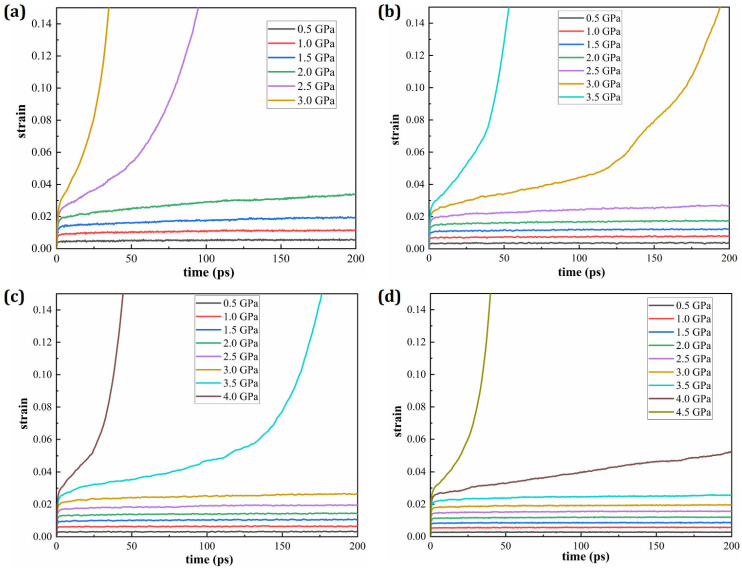
Strain responses of HESA under various stresses at constant temperatures of (**a**) 1400 K; (**b**) 1000 K; (**c**) 750 K and (**d**) 500 K.

**Figure 4:**
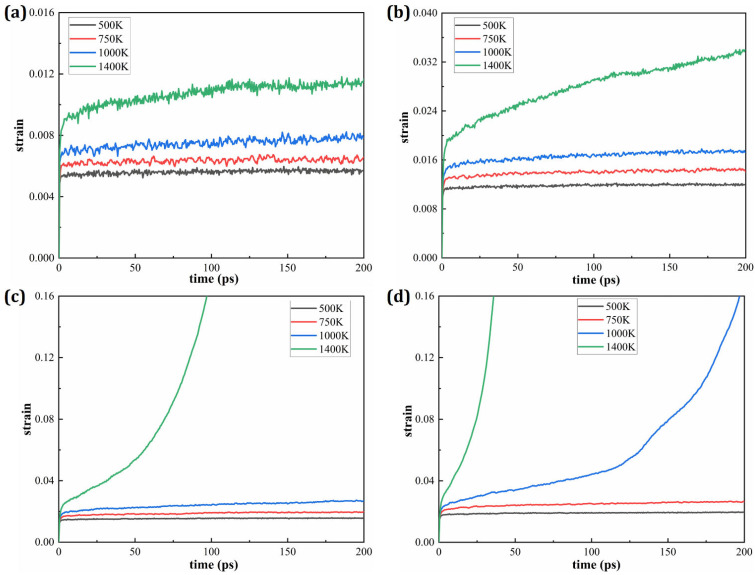
Strain responses of HESA at different temperatures under constant applied stresses of (**a**) 1.0 GPa; (**b**) 2.0 GPa; (**c**) 2.5 GPa and (**d**) 3.0 GPa.

Figure 3 presents the strain–time creep curves of HESAs under various temperature and stress conditions. Each subplot depicts the strain evolution at a fixed temperature subjected to different applied stress levels. The results clearly demonstrate that, at a constant temperature, the strain increases significantly with rising applied stress, indicating a strong stress dependence of the creep response. This further illustrates the influence of temperature on the creep behavior of HESAs. Each subplot in this figure shows the strain evolution at a constant applied stress across different temperatures. As expected, the strain increases with temperature under constant stress, confirming the pronounced temperature sensitivity of creep in HESAs. In both figures, the time intervals characterized by a nearly constant strain rate correspond to the steady-state creep stage, which is typically associated with the minimum creep rate. This stage reflects the material’s long-term creep behavior and is a critical parameter in evaluating creep resistance. It is evident that the steady-state creep rate increases with both elevated stress and temperature. Notably, a comparison of Figure 4a,d reveals that the influence of temperature on the steady-state creep rate is more pronounced than that of stress, underscoring the dominant role of thermal activation in the creep mechanisms of HESAs.

**Figure 5:**
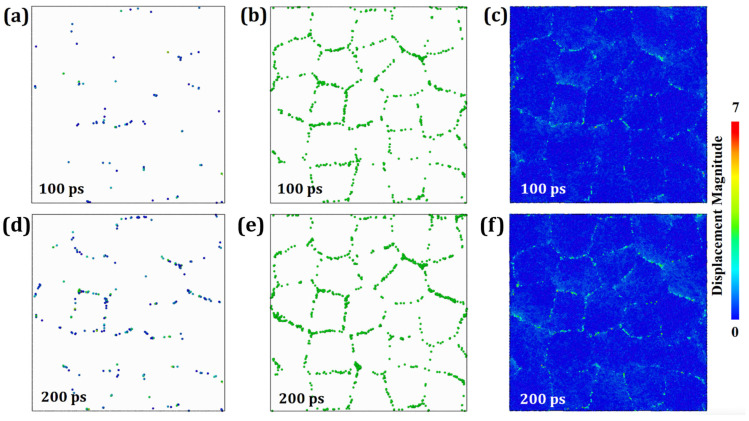
Snapshots of the HESA model at 1000 K under an applied stress of 1.5 GPa at various time intervals: (**a**,**d**) atoms with displacement greater than the nearest neighbor radius at 100 and 200 ps; (**b**,**e**) vacancy distribution at 100 and 200 ps; (**c**,**f**) atomic displacement distribution at 100 and 200 ps.

To elucidate the creep mechanism in HESA, Common Neighbor Analysis (CNA) [36], Dislocation Extraction Algorithm (DXA) [37], and Wigner–Seitz (WS) defect analysis [38] techniques are employed to investigate and visualize the defect structures. For diffusion creep, GB diffusion is considered to occur when the absolute displacement *R* of disordered atoms located at GBs exceeds the nearest-neighbor distance [35]. Figure 5 shows the results of creep analysis of HESA at 1000 K and 1.5 GPa. Figure 5 presents the results of creep simulations for HESA at 1000 K under an applied stress of 1.5 GPa. In Figure 5a, the spatial distribution of disordered atoms satisfying the criterion *R* > *R*_0_ is shown, where *R* represents the atomic displacement and *R*_0_ denotes the nearest neighbor radius (For FCC or BCC structure, the nearest neighbor radius $R_{0}=1.57a_{0}/\sqrt{2}$, where *a*_0_ is the lattice parameter). Notably, all disordered atoms are located on GBs, indicating that the GBs serve as primary diffusion pathway during creep. Figure 5b illustrates the temporal evolution of vacancy distributions, revealing that vacancies are predominantly concentrated at GBs, with virtually none observed within the grain interiors. This distribution strongly suggests that lattice diffusion is negligible under the present simulation conditions. Figure 5c depicts the atomic displacement field at various time intervals. The absence of substantial displacements at the GBs rules out the occurrence of grain boundary sliding as a dominant mechanism. Therefore, under the examined temperature and stress conditions, GB diffusion is identified as the prevailing creep mechanism in HESA. These findings further support the notion that GB diffusion occurs with significantly higher probability than lattice diffusion—consistent with previous simulation studies by Millett et al. [35], which reported higher activation energy barriers for lattice diffusion.

**Figure 6:**
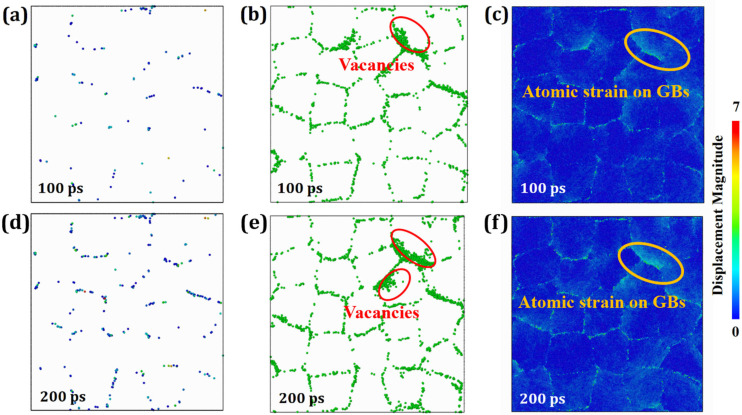
Atomic snapshots of the HESA model at 1000 K under an applied stress of 2.0 GPa at various time intervals: (**a**,**d**) atoms with displacement greater than the nearest neighbor radius at 100 and 200 ps; (**b**,**e**) vacancy distribution at 100 and 200 ps; (**c**,**f**) atomic displacement distribution at 100 and 200 ps.

**Figure 7:**
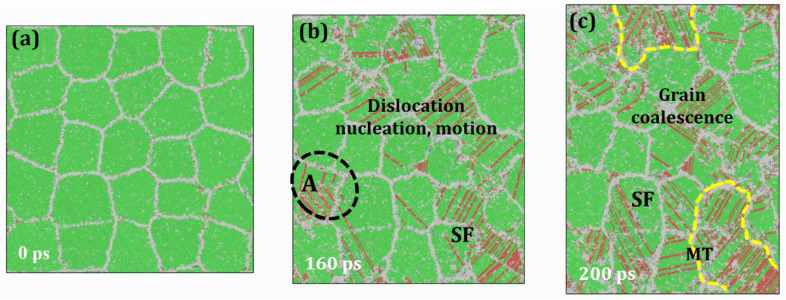
Atomic configurations of HESA at 1000 K under the applied stress of 3.0 GPa at various time intervals: (**a**) 0 ps; (**b**) 160 ps; (**c**) 200 ps.

Under a constant temperature of 1000 K, the creep mechanism of HESA evolves with increasing applied stress. When a moderate stress of 2.0 GPa is applied, as shown in Figure 6, the creep behavior exhibits notable differences compared to lower-stress conditions. Specifically, the formation of vacancies within grain interiors—highlighted by red ellipses—indicates the activation of lattice diffusion. However, the atomic displacements at grain boundaries (GBs) and within grains remain minimal and can be largely neglected. Thus, the dominant creep mechanism under this condition is identified as a combination of GB diffusion and lattice diffusion. As the applied stress increases further to 3.0 GPa, Figure 7 illustrates the resulting microstructural evolution. A significant number of dislocations are observed to nucleate at GBs and propagate into grain interiors. Over time, this dislocation activity leads to grain deformation, coalescence, and GB failure. Additionally, a stress-induced FCC-to-HCP martensitic phase transformation (MT) is detected, suggesting a more complex deformation mechanism at play. Therefore, under these specific thermomechanical conditions (1000 K and 3.0 GPa), the dominant creep mechanism is governed by a combination of dislocation nucleation, dislocation motion, and stress-induced martensitic phase transformation. In summary, as the applied stress increases at constant temperature, the creep mechanisms in HESA transition progressively from diffusion-dominated (GB and lattice diffusion) at lower stress to dislocation-mediated deformation and phase transformation at higher stress levels.

**Figure 8:**
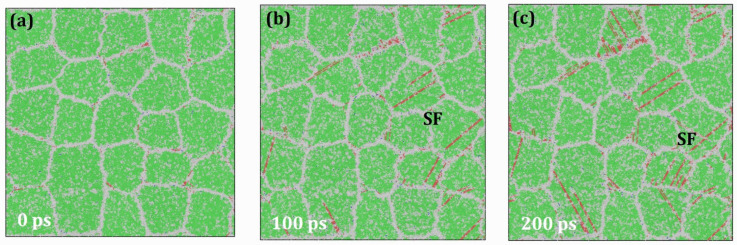
Atomic configurations of HESA at 1400 K under the applied stress of 3.0 GPa at various time intervals: (**a**) 0 ps; (**b**) 100 ps; (**c**) 200 ps.

Figure 8 presents the results of creep analysis of HESA at 1400 K under an applied stress of 2.0 GPa. It is evident that both dislocation nucleation and motion occur under these conditions. Compared to the results at 1000 K, a significant acceleration in the creep rate is observed, highlighting the strong temperature dependence of the creep behavior. Elevated temperatures not only enhance atomic mobility—leading to increased creep rates—but also substantially reduce the creep resistance of HESA. This phenomenon can be attributed to the increased atomic vibration frequency and amplitude at higher temperatures, which raises the likelihood of atoms overcoming energy barriers via thermal activation. As a result, both atomic diffusion and dislocation activity are facilitated, accelerating microstructural evolution and thereby intensifying the creep process.

### 3.2. Stress Dependence of the Stress Exponent n and Creep Mechanism

**Figure 9:**
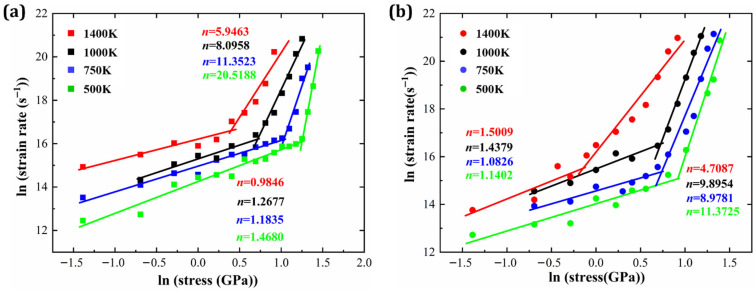
Double logarithmic plots of steady-state creep strain rate versus applied stress of (**a**) HESA and (**b**) HEA at various temperatures.

The steady-state creep behavior of alloys is typically described using the Bird–Dorn–Mukherjee equation [39]:

(4) $$\dot{\varepsilon }=\frac{AD_{0}Gb}{k_{B}T}{\left(\frac{b}{d}\right)}^{p}{\left(\frac{\sigma }{G}\right)}^{n}exp\left(-\frac{\Delta Q}{k_{B}T}\right)$$

where $\dot{\varepsilon }$ represents the steady-state creep strain rate, *A* is a dimensionless constant, *D*_0_ is the diffusion coefficient, *G* is the shear modulus, Δ*Q* is the activation energy, *k*_B_ is the Boltzmann constant, *T* is the absolute temperature, σ is the applied stress, *n* is the stress exponent, *p* is the grain size exponent, *b* is the Burgers vector, and *d* is the grain size. Among these parameters, the stress exponent *n* plays a critical role in identifying the governing creep mechanism [6,40]. Values of *n* ≈ 1–2 are typically associated with diffusion-controlled creep, whereas larger values (*n* > 3) indicate dislocation-mediated creep, and even higher values may reflect more complex mechanisms such as dislocation climb or stress-assisted phase transformation. Therefore, variations in *n* with applied stress provide a mechanistic indicator of stress-induced transitions in the dominant creep deformation mode. Based on Equation (4), Figure 9 presents log–log plots of the minimum creep strain rate versus applied stress at various temperatures. The slope of each plot corresponds to the stress exponent *n*, offering insights into the dominant deformation mechanism.

Figure 9a illustrates the stress exponent values for HESA, which span a broad range from 0.98 to 20.52. Each temperature-dependent curve in the plot can be divided into two distinct regions. Each temperature-dependent curve can be divided into two distinct stress regimes. In the low-stress regime, the extracted stress exponents fall within the diffusion-controlled range defined by Equation (4), whereas at higher stresses, the stress exponent increases markedly, indicating a transition to dislocation-dominated creep. These findings are consistent with atomic-scale observations and corroborate the proposed creep mechanism transitions. Similarly, Figure 9b shows the stress exponent n for HEA, ranging from 1.08 to 11.37. A similar stress-dependent behavior is observed for HEA, with stress exponents evolving from the diffusion-dominated regime at low stresses to dislocation-controlled deformation at higher stresses, following the same mechanistic framework as HESA. Therefore, stress-dependent creep mechanisms in HEA are essentially analogous to those observed in HESA. Furthermore, both materials exhibit a general trend in which *n* decreases with increasing temperature. This trend indicates that at elevated temperatures, the creep process becomes less sensitive to stress and more influenced by thermal activation. In such cases, thermal energy becomes the primary driving force for atomic mobility and deformation.

### 3.3. Difference in Creep Resistance Between HESA and HEA

The preceding analyses clearly indicate that under identical creep conditions, the HESA exhibits a significantly lower creep rate than the HEA, thereby demonstrating superior creep resistance. This section elucidates the underlying reasons for this difference from the perspective of microstructural evolution.

**Figure 10:**
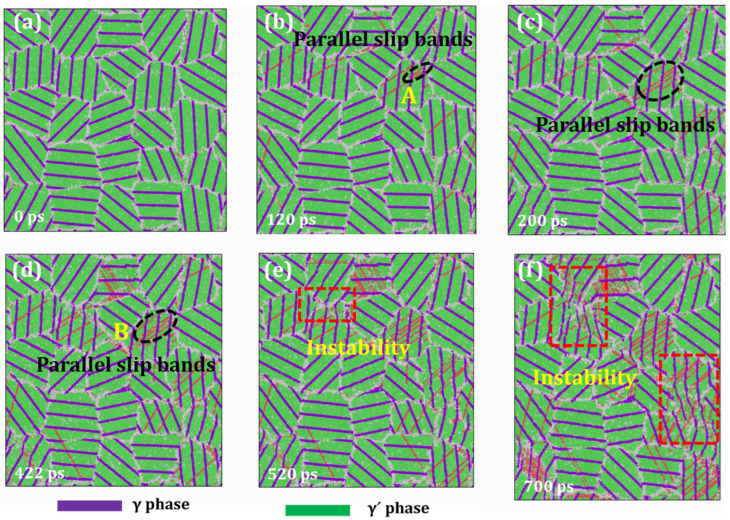
Snapshots of the creep simulation for HESA at 1000 K under an applied stress of 2.5 GPa at various time intervals: (**a**) 0 ps; (**b**) 120 ps; (**c**) 200 ps; (**d**) 422 ps; (**e**) 520 ps; (**f**) 700 ps.

Figure 10 presents the creep deformation behavior of HESA under extreme conditions (1000 K and 2.5 GPa), with distinct phase identification via color coding: the γ matrix phase is shown in purple, and the γ′ precipitates are depicted in green. The microstructural evolution reveals continuous dislocation generation at GBs, followed by their subsequent propagation into grain interiors. As creep time progresses, additional slip systems become activated, leading to concurrent dislocation motion within the same grain. This results in the formation of parallel slip bands, which are clearly observed in Figure 10. One of the key features observed is the pronounced hindering effect of the γ/γ′ interface on dislocation motion. For instance, a dislocation emitted from the GB reaches a phase boundary at *t* = 120 ps, where it is temporarily pinned by the interfacial (see position A in Figure 10b). At *t* = 422 ps, the dislocation successfully traverses the γ′ phase and reaches the next interface (position B in Figure 10d), where it experiences another pinning effect. The entire traversal process spans approximately 202 ps. This behavior underscores the critical role of phase boundaries in impeding dislocation motion. Additionally, Figure 10 highlights another important phenomenon. After substantial dislocation activity, signs of instability begin to emerge at certain grain boundaries at *t* = 520 ps (Figure 5e). However, the overall grain morphology remains stable. It is not until *t* = 700 ps (Figure 5f) that further destabilization of the phase boundaries occurs. Despite this, some grains remain unaffected, indicating no severe structural degradation at this stage. As the creep process continues, a marked acceleration in phase boundary destabilization is observed, signifying the onset of the tertiary creep regime.

**Figure 11:**
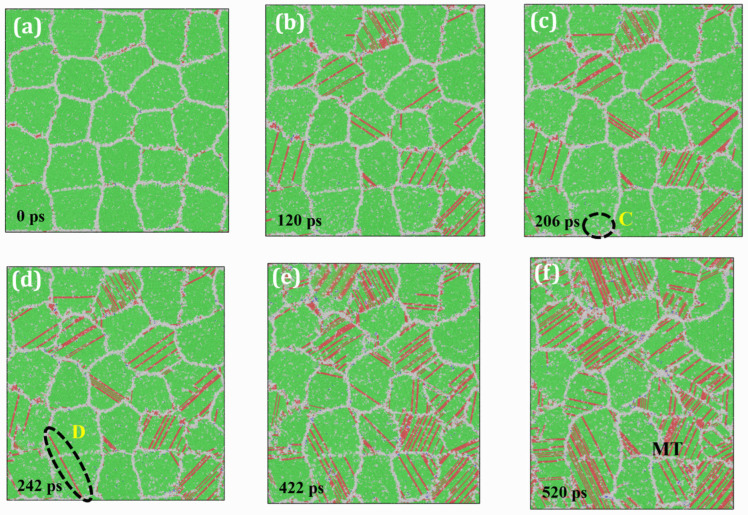
Snapshots of the creep simulation for HEA at 1000 K under an applied stress of 2.5 GPa at various time intervals: (**a**) 0 ps; (**b**) 120 ps; (**c**) 206 ps; (**d**) 242 ps; (**e**) 422 ps; (**f**) 520 ps.

In contrast, Figure 11 illustrates the creep response of HEA under the same thermomechanical conditions (1000 K and 2.5 GPa). This HEA model, composed exclusively of the γ phase, lacks the strengthening γ′ phase found in HESA. Similar to HESA, a significant number of dislocations are nucleated at GBs and propagate into grain interiors, with more slip systems being activated over time. However, a quantitative comparison reveals a stark difference in dislocation density, with HEA exhibiting a much higher dislocation density than HESA under identical creep durations. This observation is further substantiated by the rapid dislocation dynamics observed in HEA, where a representative dislocation emitted from a GB (position C in Figure 11c) completely traverses the grain and reaches the opposite GB (position D in Figure 11d) within just 36 ps. This observation reflects the reduced resistance to dislocation motion in HEA.

**Figure 12:**
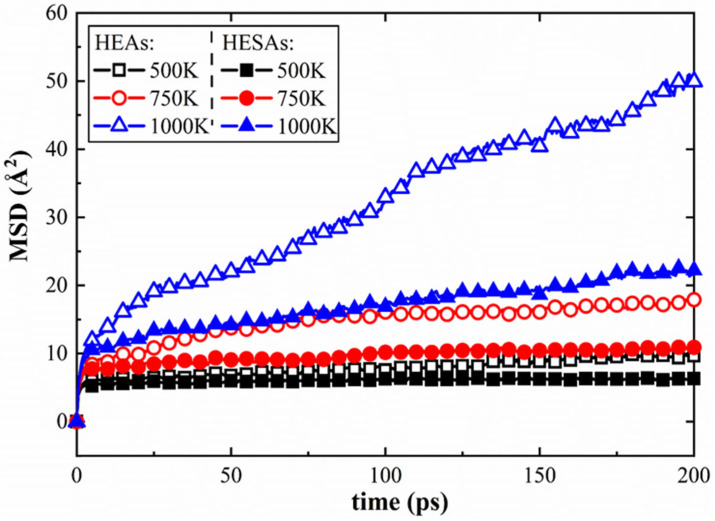
MSD values calculated for HESA and HEA at various temperatures.

Figure 12 further supports these findings by comparing the MSD values of HESA and HEA over 200 ps at various temperatures. Based on Equation (3), the diffusion coefficients (D) during the steady-state creep stage can be determined, with the corresponding results presented in Table 1. Notably, the diffusion coefficients in HEA are significantly higher than those in HESA across all temperature conditions, indicating a faster atomic diffusion rate and, consequently, a higher creep rate. In HEA, the primary barriers to dislocation motion are microstructural features such as GBs, solute atoms, and vacancies. In contrast, HESA benefits from an additional, critical obstacle—namely, the γ/γ′ phase interface. This interface not only impedes dislocation motion but also suppresses atomic diffusion, as confirmed by both microstructural observations and the lower diffusion coefficients.

In summary, the γ/γ′ interface plays a decisive role in enhancing the creep resistance of HESA. By effectively hindering dislocation motion and reducing atomic diffusion rates, it leads to a significantly lower creep rate. Therefore, the superior creep resistance of HESA compared to HEA is primarily attributed to the presence and strengthening effect of the γ/γ′ phase boundaries.

### 3.4. Effect of γ/γ′ Lattice Misfit on the Creep Properties of HESA

**Figure 13:**
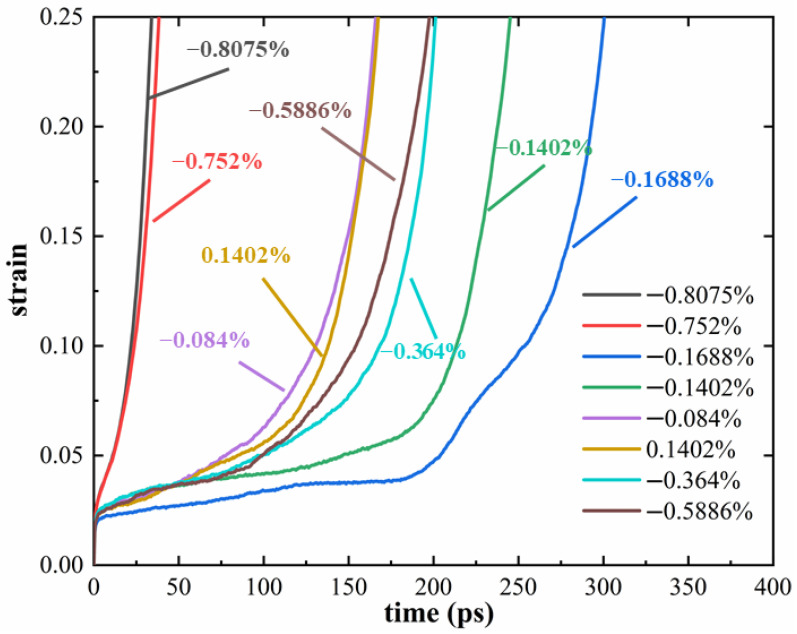
Creep curves of HESA under a stress of 3.0 GPa at 1000 K for various lattice misfit values.

The lattice parameters of the γ and γ′ phases in HESA are governed by the types and concentrations of solute atoms [17]. In conventional Ni-based superalloys, the lattice misfit between these two phases is typically negative and relatively small in magnitude (|δ| < 1%) [41]. In this study, the lattice misfit δ ranges from −0.8075% to −0.084%. To comprehensively assess the impact of lattice misfit on creep behavior, both the magnitude and sign of δ were considered. Therefore, a model with a positive lattice misfit (δ = 0.1402%) was also evaluated for comparison. The corresponding creep life results are illustrated in Figure 13. To simulate the extreme service conditions experienced by aero-engine turbine blades, a high creep temperature of 1000 K and an applied stress of 3.0 GPa were employed.

**Figure 14:**
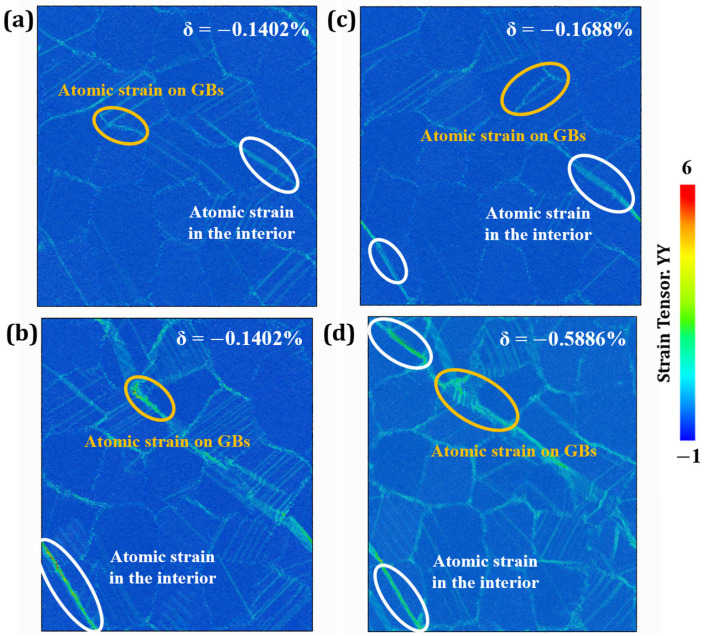
Atomic strain distribution of HESAs with lattice misfits of (**a**) −0.1402%; (**b**) 0.1402%; (**c**) −0.1688% and (**d**) −0.5886%.

As shown in Figure 13, all creep curves exhibit the typical three-stage behavior. Notably, the duration of the steady-state creep stage decreases with increasing |δ|. The relationship between creep life and |δ| is non-monotonic. Specifically, within the range of −0.1688% to −0.084%, creep life increases as |δ| increases. Conversely, within the range of −0.8075% to −0.1688%, creep life decreases as |δ| increases. The longest creep life is observed at δ = −0.1688%. To gain further insight into the underlying mechanisms, atomic strain distributions were analyzed, as shown in Figure 14. Figure 14a,b compare atomic strain maps of HESAs with negative (−0.1402%) and positive (0.1402%) lattice misfits. At *t* = 130 ps, the model with the negative lattice misfit (Figure 14a) exhibits lower atomic strain, suggesting enhanced resistance to deformation. This implies that a negative lattice misfit stabilizes the γ′ phase, strengthens the γ/γ′ interface, and impedes dislocation motion, thereby improving creep resistance. Figure 14c shows the atomic strain of HESA with δ = −0.1688% at 150 ps, further supporting the trend that moderate negative misfit enhances phase stability. This observation is consistent with the experimental findings of Zhang et al. [20]. Figure 14d extends the comparison to δ = −0.5886%, revealing that excessive negative misfit leads to increased atomic strain, indicating diminished phase stability and creep resistance. These trends are closely related to the evolution of the stress field at the γ/γ′ interface [21,42].

As has been reported, the internal stress at the phase boundary intensifies with increasing |δ|, elevating the interfacial energy and promoting a morphological transformation of the precipitated γ′ phase from ellipsoidal to cubic [43,44,45]. This transformation is driven by the misfit-induced strain energy, with δ serving as a key indicator of morphological stability. In general, a cubic morphology minimizes the alloy’s strain energy and enhances precipitate stability. However, when the value of |δ| becomes excessively large, the interfacial stress rises sharply, destabilizing the phase morphology and degrading creep resistance. This accounts for the observed non-monotonic relationship between |δ| and creep life.

**Figure 15:**
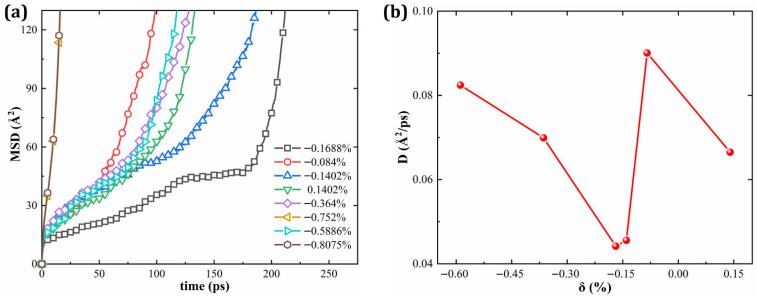
(**a**) MSD and (**b**) diffusion coefficient D of HESA as a function of lattice misfit (δ).

Figure 15a displays the MSD values calculated over 270 ps for HESAs with various lattice misfits, and Figure 15b illustrates the corresponding diffusion coefficient (D) as a function of δ. The results indicate that for δ < −0.1688%, D increases with |δ|, while in the range −0.1688% < δ < 0, D increases with decreasing |δ|. A minimum D is observed at δ = −0.1688%, corresponding to the highest creep resistance and most stable microstructure. Moreover, the comparison between D for δ = −0.1402% and δ = 0.1402% further reveals that a negative misfit yields a lower diffusion coefficient. This phenomenon aligns well with the results of the microscale analysis described earlier, further confirming the beneficial effect of a negative lattice misfit on phase stability and creep resistance.

On the whole, the influence of γ/γ′ lattice misfit on the creep behavior of HESAs parallels has been observed in conventional Ni-based superalloys. Alloys with a negative lattice misfit consistently demonstrate superior creep performance due to enhanced interface stability. When the lattice misfit is small, creep resistance improves with increasing |δ|. However, beyond an optimal range, excessive misfit impairs phase stability, thereby accelerating creep. Therefore, optimizing the lattice misfit—ensuring it remains negative and within a moderate range—is essential for improving the creep resistance and structural integrity of HESAs under high-temperature service conditions.

### 3.5. Effect of γ′ Volume Fraction on the Creep Properties of HESAs

**Figure 16:**
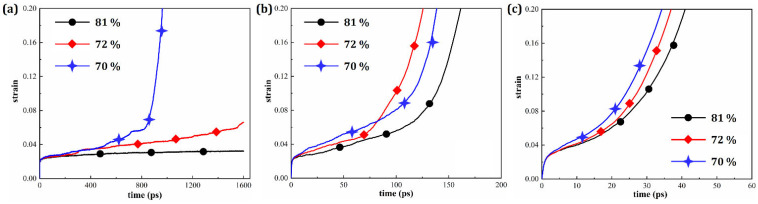
Strain evolution of HESAs with varying γ′ volume fractions at temperatures of (**a**) 750 K; (**b**) 1000 K and (**c**) 1400 K.

Figure 16 displays the creep curves for HESAs with different γ′ volume fraction (*V*_γ′_) under a constant stress of 3.0 GPa at 750 K, 1000 K, and 1400 K, respectively. It can be observed that at 750 K, the strain of the HESA with *V*_γ′_ = 81% increases slowly, indicating excellent resistance to creep deformation. For the HESA with *V*_γ′_ = 72%, the creep curve exhibits two distinct stages: primary creep and steady-state creep. In contrast, for the HESA with *V*_γ′_ = 70%, it undergoes all three typical creep stages: primary, steady-state, and tertiary creep. At higher temperatures (1000 K and 1400 K), all three alloys display the complete set of creep stages. As expected, the creep lifetime decreases with increasing temperature, which is consistent with the general trend discussed earlier. Notably, the alloy with *V*_γ′_ = 81% exhibits the longest creep life among the three, suggesting that increasing the γ′ volume fraction enhances the alloy’s resistance to creep.

**Figure 17:**
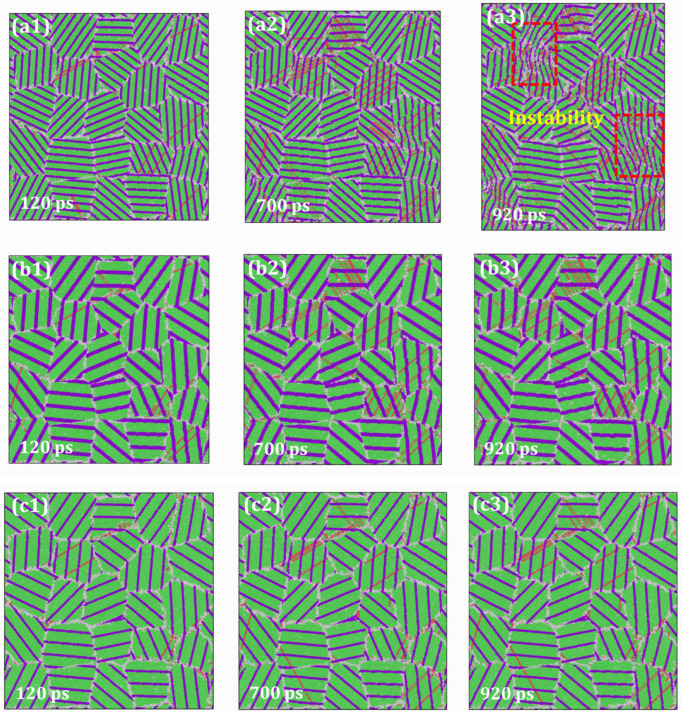
Snapshots of creep simulation for HESAs at 750 K with (**a1**–**a3**) *V*_γ′_ = 70%; (**b1**–**b3**) *V*_γ′_ = 72% and (**c1**–**c3**) *V*_γ′_ = 81%.

To further investigate the underlying mechanism, Figure 17 shows the microstructural evolution of HESAs with varying volume fractions of the precipitated phase at 750 K. At an early stage of creep (*t* = 120 ps), the number of dislocations and slip bands in HESAs with *V*_γ′_ = 70% and 72% are comparable. However, both exhibit a significantly higher density of defects compared to the HESA with *V*_γ′_ = 81%. As creep progresses (*t* = 700 ps), a notable difference in the number of defects emerges between the models with *V*_γ′_ = 70% and 72%. The model with *V*_γ′_ = 70% exhibits a higher density of dislocations and slip bands than the model with *V*_γ′_ = 72%. In contrast, the model with *V*_γ′_ = 81% consistently maintains the lowest number of defects throughout the simulation. At a later stage (*t* = 920 ps), phase boundary instability occurs in the model with *V*_γ′_ = 70%, accompanied by the destruction of partial GBs. In contrast, the phase boundaries in the models with *V*_γ′_ = 72% and 81% largely retain their initial state. The stability of the microstructure in the model with *V*_γ′_ = 81% is particularly evident, further underscoring its superior creep resistance.

**Figure 18:**
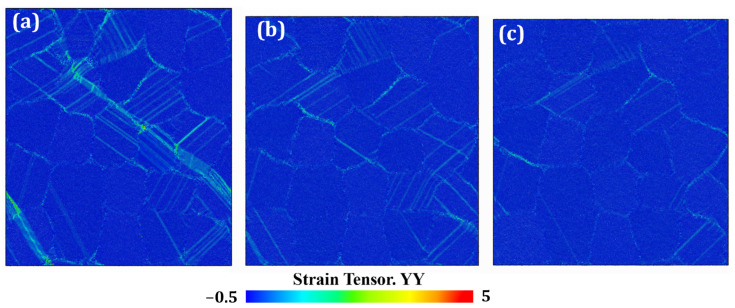
Atomic strain distributions of HESAs at *t* = 870 ps with (**a**) *V*_γ′_ = 70%; (**b**) *V*_γ′_ = 72% and (**c**) *V*_γ′_ = 81%.

Figure 18 presents the atomic strain distributions for the three alloys at *t* = 870 ps. The results clearly show that the atomic strain is highest in the model with *V*_γ′_ = 70%, moderate in the model with *V*_γ′_ = 72%, and lowest and almost negligible in the model with *V*_γ′_ = 81%. Therefore, it can be concluded that the creep resistance of the alloy is strongly correlated with the volume fraction of the precipitated phase. As the γ′ volume fraction increases, the γ/γ′ interfaces become more effective in hindering dislocation motion, while simultaneously reducing localized atomic strain. As a result, under identical creep conditions, the creep rate of the alloy decreases, leading to significantly enhanced creep resistance.

**Figure 19:**
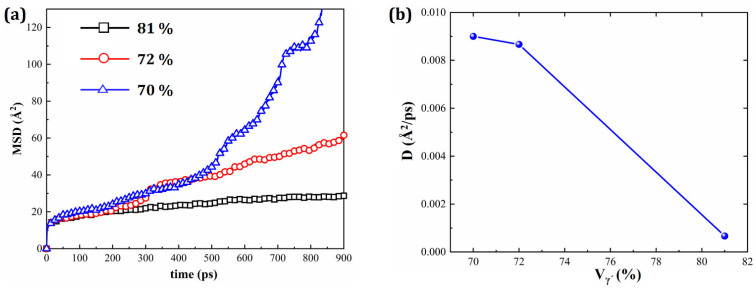
(**a**) MSD values of HESAs with varying *V*_γ′_ and (**b**) diffusion coefficient *D* versus *V*_γ′_.

To further support this observation, Figure 19a gives the MSD values calculated over 900 ps for HESAs with different γ′ volume fractions, and Figure 19b presents the corresponding diffusion coefficients. As the γ′ volume fraction increases, the diffusion coefficient decreases, indicating a reduced atomic mobility within the alloy matrix. This reduction in atomic diffusivity is beneficial to the long-term stability of the microstructure under creep conditions, and it corroborates the conclusions drawn from the strain and defect analyses.

From above, it is seen that the creep resistance of HESAs is strongly influenced by the volume fraction of the γ′ precipitated phase. The enhancement in creep performance with increasing γ′ volume fraction can be attributed to two primary mechanisms. First, the γ′ phase, being a hard and ordered intermetallic compound, serves as a strong obstacle to dislocation motion, requiring considerable stress for dislocations to bypass or cut through it. Second, a higher volume fraction of the γ′ phase reduces atomic diffusion and enhances the effectiveness of the γ/γ′ interface in impeding dislocation activity. Therefore, strategically increasing the γ′ volume fraction in HESAs offers a viable and effective approach for optimizing their creep resistance and ensuring stable high-temperature performance.

## 4. Conclusions

In this paper, MD simulations are conducted to systematically investigate the creep behavior of HESAs under varying stress, temperature, γ/γ′ lattice misfit, and γ′ volume fraction. The creep mechanism and the critical creep resistance factors are explored. The main conclusions are summarized as follows:

(1) The creep behavior of both HESAs and HEAs is strongly influenced by stress and temperature, and their combined effect governs the rate of atomic rearrangement during deformation. As these parameters increase, the creep rate accelerates rapidly due to enhanced diffusion and easier activation of deformation carriers. Temperature plays a particularly dominant role, as high thermal energy significantly facilitates atomic mobility, vacancy migration, and interface-mediated processes. Compared with HEAs, HESAs consistently exhibit lower creep strain under identical conditions, and the difference becomes more pronounced at higher stress–temperature combinations, highlighting the intrinsic microstructural advantages of γ/γ′-strengthened systems.
(2) A clear transition in creep mechanisms is observed, evolving from diffusion-dominated deformation at low stresses to dislocation nucleation, glide, and interaction at intermediate conditions, and ultimately to stress-induced phase transformation at higher stresses and temperatures. This progression is corroborated by atomic-scale structural evolution and steady-state creep theory. Under identical conditions, HESAs show markedly superior creep resistance to HEAs, mainly due to the strong blocking effect of γ/γ′ interfaces on dislocation motion and atomic transport.
(3) Both the γ/γ′ lattice misfit and γ′ volume fraction exert a critical influence on creep resistance. The relationship between misfit magnitude and creep behavior is non-monotonic: moderate negative misfit enhances interfacial coherency and stabilizes the microstructure, thereby improving creep resistance, whereas excessively large misfit increases interfacial strain energy and deteriorates performance. A negative misfit is generally more effective in resisting creep because it strengthens the γ/γ′ interface and reduces dislocation transmissibility. In addition, increasing the γ′ volume fraction significantly enhances creep resistance, as more extensive γ′ coverage reduces available diffusion pathways, suppresses defect activity, and provides more effective barriers to dislocation motion. The alloy with the highest γ′ fraction consistently exhibits the lowest creep rate and the most stable microstructural response.

In addition, the present molecular dynamics framework focuses on mechanistic insight and qualitative trends. The high stresses and strain rates reflect intrinsic timescale limitations, and the simplified two-dimensional γ/γ′ microstructure does not fully capture realistic three-dimensional precipitate morphology. Future work will address these limitations by incorporating more realistic microstructures and extended timescales.

## Figures and Tables

**Table 1:** Diffusion coefficients (*D*, in m^2^/s) of HEA and HESA at different temperatures, calculated during the steady-state creep regime using Equation (3).

	500 K	750 K	1000 K
HESA	5.5 × 10^−4^	2.75 × 10^−3^	8.87 × 10^−3^
HEA	3.32 × 10^−3^	3.82 × 10^−3^	3.1 × 10^−2^

## Data Availability

The original contributions presented in this study are included in the article. Further inquiries can be directed to the corresponding authors.
